# New insight into the electrochemical reduction of different aryldiazonium salts in aqueous solutions

**DOI:** 10.1039/d1ra04482f

**Published:** 2021-07-27

**Authors:** Zahra Tavakkoli, Hamed Goljani, Hassan Sepehrmansourie, Davood Nematollahi, Mohammad Ali Zolfigol

**Affiliations:** Faculty of Chemistry, Bu-Ali-Sina University Hamedan 65174 Iran nemat@basu.ac.ir

## Abstract

Electrochemical reduction of different aryldiazonium salts in aqueous solution was studied in this work and it is shown that the aryldiazonium salts are converted to the corresponding aryl radical and aryl anion. The results of this research indicate that the reduction of aryldiazonium salts takes place in two single-electron steps. Our data show that when the substituted group on the phenyl ring is H, Cl, OH, NO_2_, OCH_3_ or SO_3_^−^, the corresponding diazonium salt shows poor adsorption characteristics, but when the substituted group is methyl, the corresponding diazonium salt shows strong adsorption characteristics. In the latter case, the voltammogram exhibits three cathodic peaks. In addition, the effect of various substitutions on the aryldiazonium reduction was studied by Hammett's method. The data are show that with increasing electron withdrawing capacity of the substituent, the reduction of corresponding diazonium salt becomes easier.

## Abstract 2

Electrochemical reduction of different aryldiazonium salts in aqueous solution was studied. It is shown that the aryldiazonium salts are converted to the corresponding aryl radical and aryl anion.

## Graphical abstract

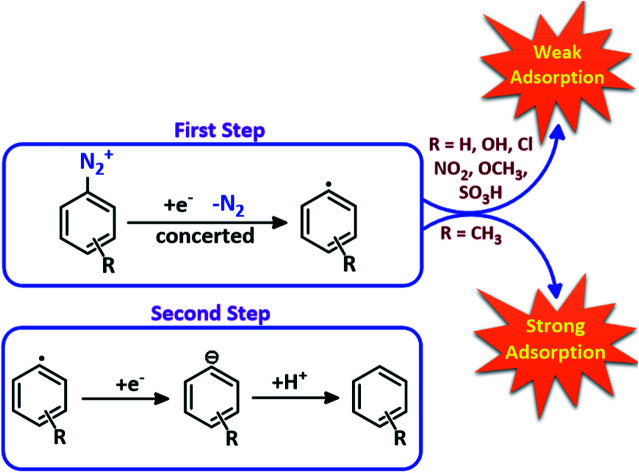

## Introduction

Diazonium salts are a large group of organic compounds with the general formula R–N_2_^+^X^−^, where R can be alkyl or aryl and X is an organic or inorganic anion such as a halogen.^1^ Diazonium salts are very important chemicals that have been used as versatile building blocks for the syntheses of a broad range of organic molecules. These compounds are able to perform two types of reactions: nitrogen-removal reactions and nitrogen-retention reactions.^2^ In the first type, with the loss of the N_2_ molecule, chemical bonds such as C–C, C–X, C–S, C–P and C–P are formed.^2–8^ In the second type, the nitrogen atoms remain in the molecule as N=N or N–N bonds.^2,5,9,10^

**Scheme 1:**
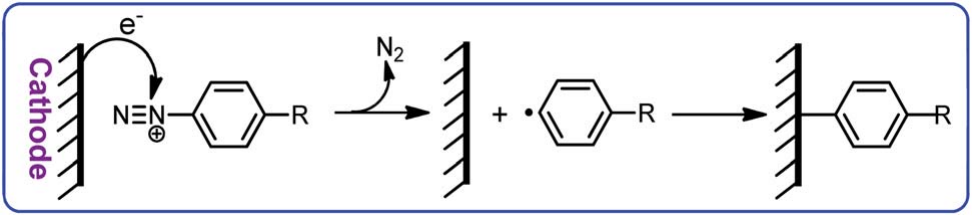
Modification of electrode surface by electrochemical grafting of diazonium salts.

The high efficiency and high selectivity of these compounds have led to an increase in their use in the synthesis of organic compounds.^11,12^ Despite the large number of published papers on the synthesis of organic compounds using diazonium salts, the electrochemical behavior of these salts has not been well studied. A literature survey on electrochemical reduction of diazonium salts show that the goal of most of these studies is the reduction of the diazonium salts to produce the corresponding radical in order to modify the electrode surface (Scheme 1).^13–21^

These studies show that the reduction of aryldiazoniums involves a single-electron transfer from the cathode to the aryldiazonium salt, resulting in the release of a nitrogen molecule and the formation of an aryl radical followed by the bond formation between the electrode surface and the aryl group. Many researchers have worked on the grafting of aryldiazonium salts on different electrode surfaces, and obtained satisfactory results. Among them we can mention the interesting studies conducted by Pinson^22–31^ and Downard *et al.*^32–41^ In these published papers, the authors provided valuable information on the electrochemical reduction mechanism of aryldiazoniums in acetonitrile. Nevertheless, this study seeks to expand the frontiers of knowledge on some electrochemical properties of aryldiazonium salts in aqueous solutions. Therefore, in this paper, we want to provide some new information on the electrochemical behavior of aryldiazonium salts in water and investigate the effect of substituent groups on adsorption activity and electrochemical behavior of these compounds. This study will contribute to expand the understanding of electrochemical reduction of aryldiazonium salts in aqueous solutions.

## Results and discussion

### Mechanistic studies

**Fig. 1:**
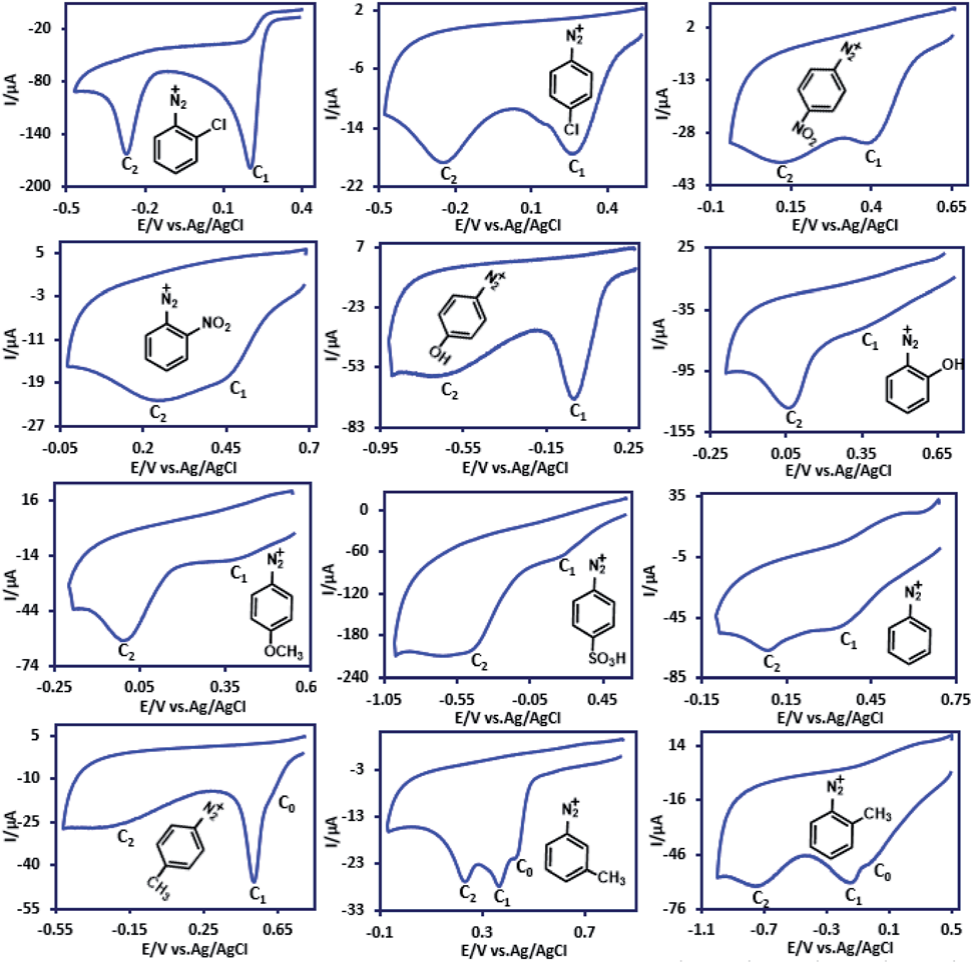
Cyclic voltammograms of 10 mM of ADs in aqueous solution (H_2_SO_4_, *c* = 1 M) at glassy carbon electrode. Scan rate: 100 mV s^−1^. Temperature: 4 ± 1 °C.

Cyclic voltammograms of twelve aryldiazonium hydrogen sulfate salts (ADs) (10 mM) containing H, Cl, NO_2_, OCH_3_, SO_3_^−^, OH and CH_3_ substituent groups in aqueous solution (H_2_SO_4_, *c* = 1.0 M) at a temperature 4 ± 1 °C are shown in Fig. 1. The first common feature of these diazonium salts is their irreversibility. The absence of anodic peak even at high potential scan rates indicates the high reactivity of the electrode product and its participation in an irreversible fast chemical reaction. Most of these compounds (containing Cl, NO_2_, OCH_3_, SO_3_H, H and OH substituent groups) have two cathodic peaks (C_1_ and C_2_) and some (containing CH_3_ substituent group), have three cathodic peaks (C_0_, C_1_ and C_2_).

**Fig. 2:**
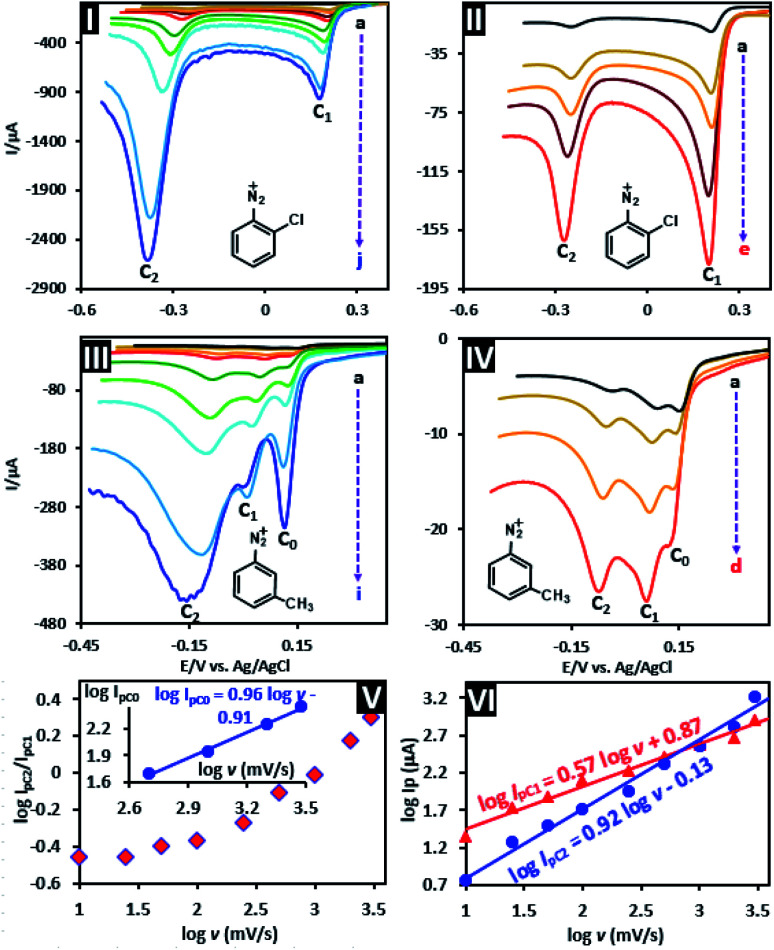
Parts I and II: linear sweep voltammograms of 10 mM of 2ClAD. Parts III and IV: linear sweep voltammograms of 10 mM of 3MeAD in aqueous solution (H_2_SO_4_, *c* = 1 M) in various scan rates at glassy carbon electrode. Scan rates from (a) to (j) are: 10, 25, 50, 100, 250, 500, 1000, 2000, 3000 and 4000 mV s^−1^. Part V: plot of log *I*_pC_2__/*I*_pC_1__ values derived from linear sweep voltammograms of 2ClAD (parts I and II) *vs.* log *v*. Part VI: plot of log *I*_pC_1__ and *I*_pC_2__ values derived from linear sweep voltammograms of 2ClAD (parts I and II) *vs.* log *v*. Part V, inset: plot of log *I*_pC_0__ values derived from linear sweep voltammograms of 3MeAD (parts III and IV) *vs.* log *v*. Temperature: 4 ± 1 °C.

To explain this difference, the linear sweep voltammograms of 2-chlorobenzenediazonium (2ClAD) and 3-methylbenzenediazonium (3MeAD) were recorded at a temperature 4 ± 1 °C, at different scan rates (Fig. 2). As can be seen, in both compounds the peak current ratio (*I*_pC_2__/*I*_pC_1__) changes with increasing scan rates. This shows the unequal influence of the adsorption process on two single-electron transfer steps. The examination of 2ClAD voltammograms shows that while at a scan rate of 10 mV s^−1^, the peak current ratio, *I*_pC_2__/*I*_pC_1__ is about 0.3 (log *I*_pC_2__/*I*_pC_1__ = −0.52), with the increase of potential scan rate to 4000 mV s^−1^, the ratio increases sharply to about 2.4 (log *I*_pC_2__/*I*_pC_1__ = 0.38). These results indicate that the adsorption ability of the product of second electron transfer process is more than that of the first electron transfer process. To confirm this statement, the dependence of logarithm of the C_1_ and C_2_ peak currents (log *I*_pC_1__ and log *I*_pC_2__) on the logarithm of potential scan rate (log *ν*) were examined (Fig. 2, part VI). Under these conditions, the slope of the line for a pure diffusion controlled process is 0.5,^42^^(p. 236)^ while this value is equal to 1 for a pure adsorption process.^42^^(p. 591)^ The slope of log *I*_pC_1__*vs.* log *ν* is 0.57. This value is higher than the theoretical value of 0.5 and is less than one, which indicate a partial adsorption for peak C_1_. The slope of log *I*_pC_2__*vs.* log *ν* is 0.92. This value is near to one which is the theoretical value for the adsorption-controlled process and confirms the greater adsorption ability of the product of second electron transfer process than that of the first electron transfer process.

The voltammograms of 3MeAD however, show a more complex behavior. The linear sweep voltammogram of 3MeAD at a scan rate of 10 mV s^−1^, shows three cathodic peaks (C_0_, C_1_ and C_2_). At low scan rates, *I*_C_0__ and *I*_C_1__ are larger than the *I*_C_2__. As the potential scan rate increases, *I*_C_0__ increases linearly and its shape (peak C_0_) becomes sharper and more symmetrical. Under these conditions, the slope of log *I*_pC_0__*vs.* log *ν* is 0.96 (Fig. 2, part V, inset). This value confirms that peak C_0_ is an adsorption peak which is separated by a 96 mV from peak C_1_. This type of peak is observed when the electrode product is strongly adsorbed.^42^^(p. 596)^ The response of peaks C_1_ and C_2_ to increasing potential scan rate is generally similar to that seen for 2ClAD.

**Fig. 3:**
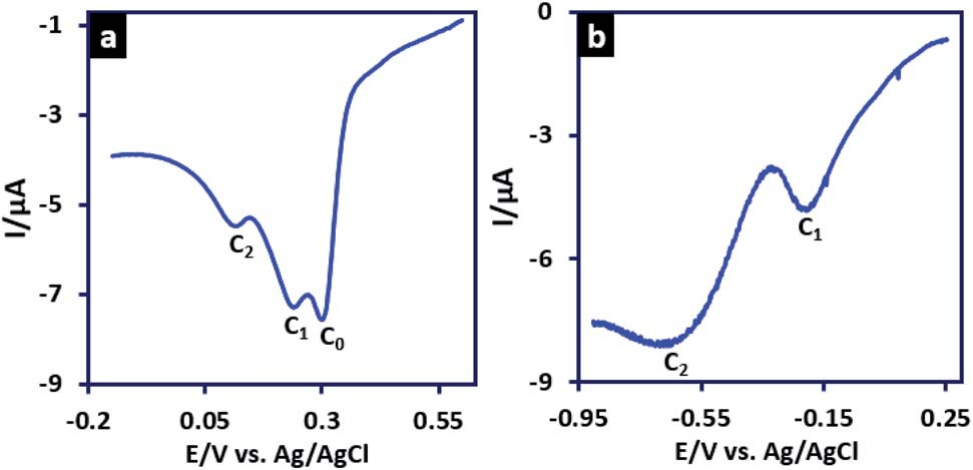
(a) LSV of 3MeAD (10 mM) in aqueous solution (H_2_SO_4_, *c* = 1 M) and (b) LSV of 3MeAD (10 mM) acetonitrile solution (HClO_4_, *c* = 1 M). Scan rate: 10 mV s^−1^. Temperature: 4 ± 1 °C.

The LSV of 3MeAD in aqueous solution (H_2_SO_4_, *c* = 1 M) is shown in Fig. 3a and has been compared with that of in acetonitrile solution containing HClO_4_ (1 M) (Fig. 3b). The most important feature of Fig. 3b is the presence of two peaks. Based on our findings on the adsorption nature of peak C_0_, this change in acetonitrile solution was predictable. The polarity of the solvent has a significant effect on the adsorption of organic compounds onto electrode surface. The higher solubility of the organic compounds in organic solvents decreases the adsorption process due to the higher affinity between organic compound and solvent.^43^ Therefore, in acetonitrile solution, peak C_0_ (a pre-peak) is removed and the voltammogram shows only peaks C_1_ and C_2_.

The presence of two peaks in the voltammograms of aryldiazoniums have also been reported in other studies.^25,31,44–48^ Downard and coworkers studied the electrochemical reduction of 4-nitrobenzenediazonium ion in [Bu_4_N]BF_4_–ACN and stated that the presence of two peaks in voltammograms is due to “*a surface-catalyzed reduction step (proceeding at a clean surface only) followed by an uncatalyzed reduction at a more negative potential*”.^48^ Since our studies were conducted in aqueous solution (H_2_SO_4_, *c* = 1.0 M), but Downard's research in [Bu_4_N]BF_4_–ACN, there are some important differences in our results. In the voltammograms recorded by Downard *et al.*,^48^ the ratio of the first cathodic peak to the second cathodic peak (*I*_pC_1__/*I*_pC_2__) increases with increasing potential scan rate, while our results are completely opposite (Fig. 2). Also in the case of some aryldiazonium salts such as 3MeAD, we clearly observed three cathodic peaks (C_0_, C_1_ and C_2_) while such a case was not reported by Downard *et al.*

In connection with the observation of two cathodic peaks in the cyclic voltammogram of aryldiazonium salts, Pinson *et al.* reported that (in [Bu_4_N]BF_4_–ACN) the steric effect can limit or even suppress aryl radical grafting for aryldiazonium substituted in *ortho* position.^49^ They reported that the cyclic voltammograms under these conditions show only one cathodic peak.^49^ However, we observed that, the position of the substituted group on the phenyl ring has no effect on the number of peaks. As shown in Fig. 1 and 2, the cyclic voltammograms of aryldiazonium salts such as 2ClAD, 2MeAD and 2NO_2_AD clearly show the presence of two cathodic peaks.

In this regard, Richard and co-workers investigated the electrochemical reduction of 4-nitrobenzenediazonium ion in water (0.1 M HCl) at GC electrode and announced that peak C_1_ corresponds to the reduction of 4NO_2_AD with concomitant grafting, and peak C_2_ corresponds the reduction of the grafted nitro group.^46^ This statement cannot be general, because, as shown in Fig. 1, there are two peaks in the cyclic voltammogram of aryldiazonium salts with other substituent groups, such as CH_3_, Cl, H or OH.

**Scheme 2:**
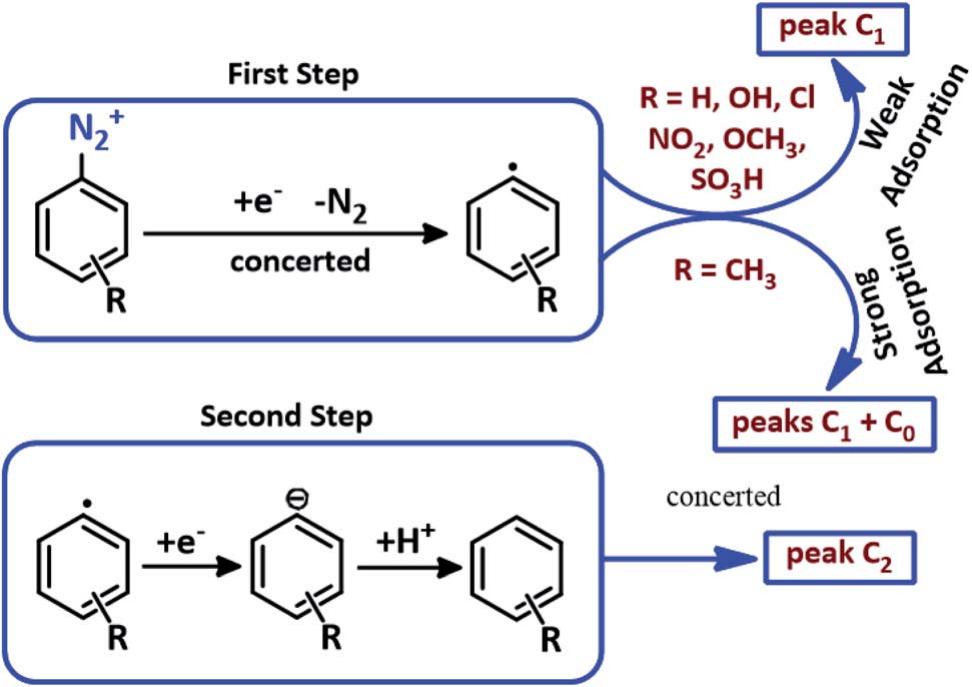
Electrochemical reduction pathway of ADs.

Based on our results, it is clear that the data reported for the presence of two reduction peaks in acetonitrile solution cannot be used in water. According to our results, it can be concluded that in the case of 3MeAD, we are confronted with the strong adsorption of electrochemically generated aryl radicals. This strong adsorption causes peak C_0_ to appear as a pre-peak in the CVs of 3MeAD.^42^ These data, along with the results of previously published results^50^ can be used to propose the following pathway for the electrochemical reduction of ADs (Scheme 2). According to the proposed scheme, in the first stage an electron transfer concerted with the cleavage of dinitrogen^50^ produces the aryl radical and in the second stage, the produced aryl radical is converted to related aromatic compound by taking one electron and one proton.

### Hammett studies

The effect of substituents on the reduction potential of AD compound is analyzed using the Hammett equation:^51^

**Fig. 4:**
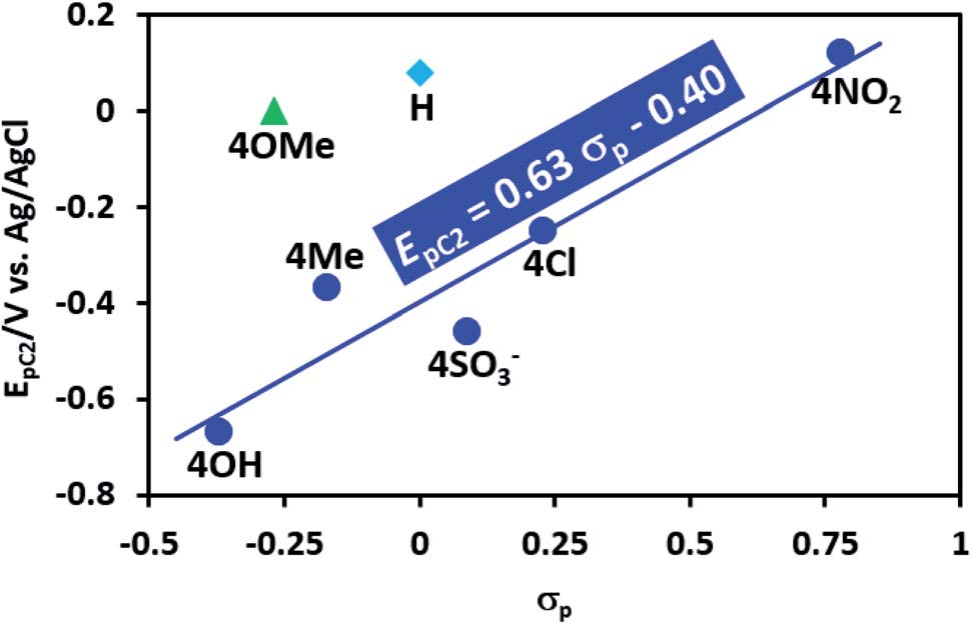
Relationship between log *E*_pC_2__ and *σ*_p_ for AD derivatives in aqueous solution (H_2_SO_4_, *c* = 1 M) at glassy carbon electrode. Temperature: 4 ± 1 °C.

1 log *E*_i_ = log *E*_0_ + *ρσ*_p_

where, *E*_i_ is the reduction potential of substituted AD, *E*_0_ is the reduction potential of AD when R = H, *σ* is the substituent constant (Hammett constant), which is dependent on the substituent group (in *para* position) and *ρ* is the slope of the log *E*_i_–*σ* graph, reflecting the sensitivity of reduction potentials to the substituent effects. The Hammett plot for AD compounds is shown in Fig. 4. As can be seen, there is a relatively good linear relationship between reduction peak potential (*E*_pC_2__) and Hammett constant (*σ*). The slope of the line is 0.63. The positive slope indicates that the reduction potential of AD compounds is increased (easier reduction) significantly with increasing electron-withdrawing ability of the substituents. The results also show that the reduction potential of AD compounds to vary in the order 4NO_2_AD (*σ*_p_ = 0.78 (ref. 52)) > 4ClAD*σ*_p_ = 0.23 (ref. 52)) > 4SO_3_^−^AD (*σ*_p_ = 0.09 (ref. 53 and 54)) > 4MeAD (*σ*_p_ = −0.17 (ref. 52)) > 4OHAD (*σ*_p_ = −0.37 (ref. 52)). On the other hand, 4-methoxybenzenediazonium (4MeOAD) has a significant deviation from *E*_pC_2__–*σ*_p_ plot. This can be due to the dual properties OCH_3_ group (resonance and inductive). The methoxide is inductively a withdrawing and resonantly a donating group. At the moment, we do not have a strong argument for diverting HAD from the *E*_pC_2__–*σ*_p_ plot. This linear relationship shows that reduction pathway of mentioned substituents are same together.

## Conclusions

In this study electrochemical reduction of AD derivatives has been studied in aqueous solution (H_2_SO_4_, *c* = 1 M) and in temperature 4 ± 1 °C by cyclic voltammetry technique at a glassy carbon electrode. Our results show two irreversible cathodic processes which are attributed to the reduction of ADs to the corresponding aryl radical and reduction of aryl radical to aryl anion. Our data indicate that when the substituted group on the phenyl ring is H, Cl, OH, NO_2_, OCH_3_ or SO_3_^−^, the corresponding diazonium salt shows poor adsorption characteristics. As a result, the voltammograms of these diazonium salts show only two reduction peaks (C_1_ and C_2_). But when the substituted group is methyl, the corresponding diazonium salts show strong adsorption characteristics. Therefore, the voltammograms of these diazonium salts, in addition to the peaks C_1_ and C_2_, show the adsorption peak C_0_. The effect of substituent groups on the reduction of ADs was investigated by Hammett studies. The data are show that with increasing electron withdrawing capacity of the substituent, the reduction of corresponding diazonium salt becomes easier. In the end, according to Downard^48^ “*the origin of this intriguing behavior remains controversial*”.

## Experimental section

### Apparatus and reagents

Cyclic voltammetry and linear sweep voltammetry were performed using an Autolab model PGSTAT 20 potentiostat/galvanostat equipped with NOVA 1.10 software. The working electrode used in the voltammetry experiments was a glassy carbon disc (2.6 mm diameter) and a platinum wire was used as the counter electrode. The working electrode potentials were measured *vs.* Ag/AgCl (3.0 mol L^−1^ KCl) (all electrodes from AZAR electrodes). The glassy carbon electrode was polished using alumina slurry followed by washing with water and acetone. All anilines derivatives were obtained from commercial sources.

Aryldiazonium salts were synthesized by adding 0.1 mmol aniline derivatives in 10 ml aqueous solution containing 1.0 M of sulfuric acid. After cooling the temperature of the solution down to 4 ± 1 °C, 0.12 mmol of sodium nitrite was added to the mixture. In order to synthesize aryldiazonium salts suitable for study in organic solvents, follows the method already described and use only perchloric acid instead of sulfuric acid. The precipitate was separated by filtration and washed several times with cold distilled water. In the following, for voltammetry in acetonitrile, 0.1 mmol of the precipitate was dissolved in 10 ml acetonitrile containing 1.0 M perchloric acid.

## Conflicts of interest

The authors declare no conflict of interest.

## Supplementary Material
